# Exploring the Relationships Between Yield and Yield-Related Traits for Rice Varieties Released in China From 1978 to 2017

**DOI:** 10.3389/fpls.2019.00543

**Published:** 2019-05-07

**Authors:** Ronghua Li, Meijuan Li, Umair Ashraf, Shiwei Liu, Jiaen Zhang

**Affiliations:** ^1^College of Natural Resources and Environment, South China Agricultural University, Guangzhou, China; ^2^Key Laboratory of Agro-Environment in the Tropics, Ministry of Agriculture, South China Agricultural University, Guangzhou, China; ^3^Department of Botany, University of Education (Lahore), Faisalabad-Campus, Faisalabad, Pakistan; ^4^College of Agriculture, South China Agricultural University, Guangzhou, China

**Keywords:** correlation coefficient, hybrid, inbred, rice, yield and yield components

## Abstract

Despite evidence from previous case studies showing that agronomic traits partially determine the resulting yield of different rice (*Oryza sativa* L.) varieties, it remains unclear whether this is true at the ecotype level. Here, an extensive dataset of the traits of 7686 rice varieties, released in China from 1978 to 2017, was used to study the relationship between yield and other agronomic traits. We assessed the association between yield and other agronomic traits for four different rice ecotypes, i.e., indica inbred, indica hybrid, japonica inbred, and japonica hybrid. We found that associations between agronomic traits and yield were ecotype-dependent. For both the indica inbred and indica hybrid ecotypes, we found that greater values of certain traits, including the filled grain number per panicle, 1000-grain-weight, plant height, panicle length, grains per panicle, seed setting rate, long growth period, low panicle number per unit area, and low seed length/width ratio, have accounted for high grain yield. In the japonica inbred and japonica hybrid ecotypes, we found that only high panicle number per unit area and long growth period led to high grain yield. Indirectly, growth period consistently had a positive effect on yield in all ecotypes, and plant height had a positive effect on yield for the indicas and japonica inbred only. Plant height had a negative effect for the japonica hybrid. Altogether, our findings potentially have valuable implications for improving the breeds of rice ecotypes.

## Introduction

Rice (*Oryza sativa* L.) is one of the oldest and most important cereal crops—it has been cultivated for 8,200–13,500 years (Molina et al., 2011). Rice is the staple food of 60% of China’s population and more than 50% of the global population. In 2017, the rice production of China comprised 28.8% of the total global rice production^1^. Compared with the average global rice production of 4.3 t ha^-1^, the average yield of rice in the 30 million ha of China^2^ is 6.8 t ha^-1^. However, the increasing frequency of temperature extremes, onset of droughts, storms and floods, a rapidly growing population and urbanization are still major constraints against ensuring food security (Foley et al., 2011; Ray et al., 2012; Qian et al., 2016; Zeng et al., 2017). Therefore, it is essential to develop high-yielding, climate-resilient and high-quality rice varieties (Venu et al., 2011; Zeng et al., 2017). The development of semi-dwarf varieties through heterosis has substantially increased rice yield in the past 50 years (Xing and Zhang, 2010; Qian et al., 2016). Recently, a rational design approach, which is based on extensive accumulated knowledge about the genes that regulate important agronomic traits, has been re-proposed to increase the accuracy and effectiveness of genetic selection for pyramiding multiple desirable traits (Qian et al., 2016; Zeng et al., 2017).

Yield is one of the most important and complex traits in rice. It is both regulated by genes known as quantitative trait loci and influenced by external environmental factors (Wang et al., 2012; Zeng et al., 2017; Zhang L. et al., 2017). In rice, yield is determined by indirect traits like plant height, growth period, tillering ability, panicle length, seed length, seed setting rate, and grains per panicle as well as direct traits like panicle number per unit area and/or per plant, filled grains per panicle and 1000-grain-weight (Moldenhauer and Nathan, 2004; Sakamoto and Matsuoka, 2008; Huang et al., 2013).

There are more than 40,000 rice varieties in the world and more than 15,000 of them have been cultivated throughout China^3^. Asian cultivated rice has experienced significant genetic differentiation by adapting to different ecological conditions under both natural and human selection. This genetic differentiation has generated a wealth of genetic diversity in rice, such as indica and japonica ecotypes (Vaughan et al., 2008; Lu et al., 2009). During the process of domestication, the indica and japonica rice varieties have diverged in morphological characteristics and agronomic, physiological and biochemical features, as well as in yield, quality and stress resistance. The indica and japonica ecotypes can be further divided into inbred and hybrid varieties, so there are four main types, i.e., indica inbred (II), indica hybrid (IH), japonica inbred (JI), and japonica hybrid (JH).

Indica rice is mainly cultivated in tropical and subtropical areas at low altitudes, including the Yangtze River region and south coast region, and accounts for 70% of the total rice field area in China (Wang et al., 2015). Japonica rice is more cold-tolerant than indica rice, so it is cultivated in areas with a larger temperature range, often at higher latitudes or altitudes, including the northeast region and south-western plateau region with higher altitude in China. The japonica rice growing area has increased over time, to about 9 million ha, and thus accounts for 30% of the total rice area in China. Hybrid rice is extensively cultivated in more than 53% of the total rice area in China, with 51.5% IH and 1.5% JH (Maclean et al., 2013).

It is important that future research focuses on understanding the nature and strength of the relationship between yield and its components, which will improve the efficiency of genetic selection in plant breeding programs. Researchers have already quantified associations between yield and yield components in a number of studies (Gravois and Helms, 1992; Casanova et al., 2002; Chung et al., 2005; Zahid et al., 2006; Chandra et al., 2009; Huang et al., 2011). However, most of them used a small sample size, and no detailed assessment of the associations have been attempted to date. To consider the yield and yield components of direct (e.g., panicle number per unit area, filled grain number per panicle, and 1000-grain-weight) and indirect traits (e.g., growth period, plant height, panicle length, grains per panicle) (Moldenhauer and Nathan, 2004; Sakamoto and Matsuoka, 2008; Huang et al., 2013), the present study was conducted using a dataset of rice traits from varieties released in China. We used this extensive dataset to assess how the variation in final yield is associated with the differences in agronomic traits among the four rice ecotypes (II, IH, JI, and JH). Additionally, a path analysis was also conducted to reveal the direct and indirect effects on trait-yield relationships for the four types of rice cultivars. Specifically, we used a dataset of 7,686 rice varieties, which accounts for 51% of the rice varieties used in breeding and/or cultivation in China, and quantifies several key traits to test the associations between yield and its components.

## Materials and Methods

In China, new rice varieties must be registered with the provincial or national crop variety registration committees before release. To identify elite rice varieties, the government of China established an evaluation system for new rice varieties in the 1960s. First, new breeding lines are observed by breeders in comparison trials at breeding stations over a 1–2 years period. Next, the most promising lines are recommended for the provincial or national rice variety regional trial. The regional trial is conducted by the government crop variety administration department. Regional trials are performed at 10–20 test sites in the rice ecosystems of the region where the variety was selected. At each test site, the trial is carried out by using a randomized complete block design with three replications. Each plot is 13.3 m^2^. Agronomic traits such as yield, yield components, growth period, plant height, seed length, seed width, adaptability, stability, resistance to main diseases and pests and grain quality are evaluated for each line. After completing 2–3 years of regional trials, new varieties are submitted to a provincial or national crop variety registration committee for registration (Yang et al., 2006). Our data originates from the 2–3 year-long regional trials. Grain yield was determined for each 13.3 m^2^ plot and adjusted to a standard moisture content of 0.135 g H_2_O g^-1^ fresh weight for indica ecotypes and to 0.145 g H_2_O g^-1^ fresh weight for japonica ecotypes. At maturity, plant samples were collected randomly across ten hills, except for two border rows at the side of each plot. On each hill, panicles with five or more grains were counted to determine the panicle number per ha. Other measurements were also taken on each hill. Plant height was measured from the ground surface to the tip of the tallest panicle. The length of each panicle was measured from neck to tip. The number of grains was counted for each panicle to measure grains per panicle. The number of grains that were filled more than 30% was determined to measure filled grain number per panicle. Seed setting rate was calculated as the ratio of filled grain number per panicle to grains per panicle. Fully filled grains were used for measuring grain width, length and length/width ratio. After drying in the air, 1000-grain-weight was calculated based on 1,000 grains in each plot. Growth period was measured from sowing until maturity.

This trait information for all the rice varieties was collated from previously published reports and articles, from the China Rice Data Center^4^ and the Chinese Crop Germplasm Resources Information System^5^. The China Rice Data Center^4^ is a database of agronomic traits and pedigree information for rice varieties released by provincial or national seed boards in China. The website was built and is maintained by the China National Rice Research Institute of the Chinese Academy of Agricultural Sciences. The Chinese Crop Germplasm Resources Information System^5^ is a web site built by the Institute of Crop Science of the Chinese Academy of Agricultural Sciences. The database^5^ contains agronomic traits of 13944 rice varieties. However, there is no information for yield or year of release for some of these varieties. Thus, for some rice varieties, we combined data from the two websites and from published reports and articles. The trait-related database covers rice varieties released by provincial or national seed boards between 1978 and 2017 in China (Supplementary Data Sheet S1). The collated data included grain yield (GY), panicle number per unit area (PN), filled grain number per panicle (FGN), 1000-grain-weight (TGW), growth period (GP), plant height (PH), panicle length (PL), grains per panicle (GPP), seed setting rate (SS) and seed length/width ratio (LW). The rice varieties were divided into four ecotypes: indica inbred (II, 767 varieties), indica hybrid (IH, 4814 varieties), japonica inbred (JI, 1809 varieties) and japonica hybrid (JH, 296 varieties). In this study, we did not include the indica-japonica hybrid because few varieties have been released.

It should be noted that there are missing trait values in the dataset for some varieties. Less than 10% of values for GP, PH, and TGW are missing, while 13.8% of SS are missing. About 30% of PN, FGN, PL and LW are missing, and 42% of values for GPP are missing.

### Data Analysis

We examined whether individual trait values differed among the four rice ecotypes using one-way analysis of variance (ANOVA). Bivariate trait relationships were summarized with Pearson correlations and with standardized major axis (SMA) slopes. We used standardized major axis regression to test if the regression coefficients between GY and other agronomic traits differed significantly among ecotypes (Warton et al., 2012; Michaletz et al., 2014). Before the SMA analysis, variables were standardized to have a mean of 0 and a standard deviation of 1. Pair-wise comparisons were made between every two ecotypes to test differences in their slopes. We noted that the *R*^2^ and *P*-values of an SMA relationship are identical to those of an ordinary least squares regression. Also, the *P*-value of an SMA relationship is identical to that of Pearson correlation. The analysis was carried out using sma() from the R package “smatr,” and we provide detailed script for the analysis in Supplementary Table S1.

We constructed a structural equation model (SEM) to quantify the multivariate causal network in which GY, PN, FGN, TGW, GP, and PH were all involved. PN, FGN and TGW were considered direct factors, while GP and PH were considered indirect. Structural equation modeling is a multivariate statistical analysis technique that is used to analyze structural relationships (Grace and Bollen, 2005). This technique can be viewed as the combination of factor analysis and multiple regression analysis. It estimates the multiple and interrelated dependence in a single analysis. Note that the undirected relationships (double-headed arrows) represent the covariances among variables in a model. While the coefficients associated with directed paths between two variables are partial regression coefficients (note the coefficient does not represent bivariate correlation between the two variables). In short, partial regression represents a method of statistical control that removes the effect of correlated influences. Because so many values were missing, PL, GPP, SS, and LW were not included in the SEM. To make coefficients of different pathways comparable, all selected variables were standardized to have a mean of 0 and a standard deviation of 1 before the SEM analysis. We used the R software platform (R Core Team, 2015) and the lavaan package (Rosseel, 2012) for our structural equation model analyses. If one of the varieties contained missing values, we used pairwise deletion to handle the missing data. We used the adjusted goodness-of-fit statistic (AGFI > 0.90) and the standardized root mean square residual (SRMR < 0.05) to evaluate overall model fit (Hooper et al., 2008). We provide a detailed script for the analysis in Supplementary Table S1.

## Results

### General Differences in Trait Values Among Rice Ecotypes

**FIGURE 1:**
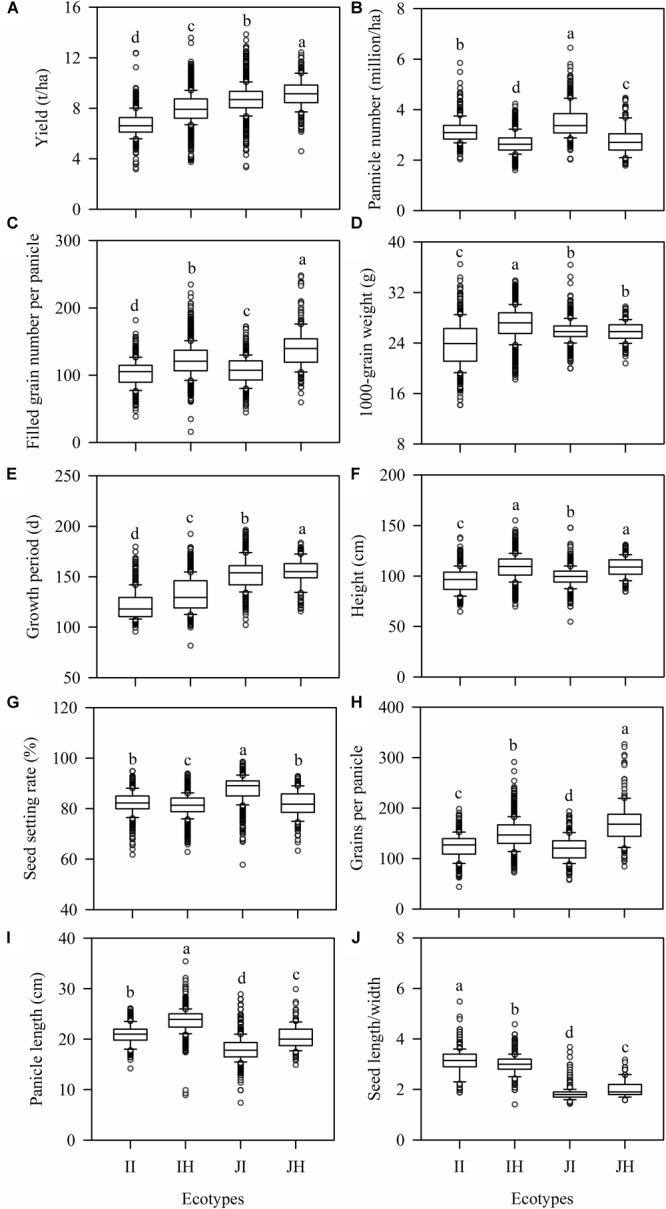
Differences in trait values among rice ecotypes. **(A)** yield, **(B)** panicle number per unit area, **(C)** filled grain number per panicle, **(D)** 1000-grain-weight, **(E)** growth period, **(F)** plant height, **(G)** panicle length, **(H)** grains per panicle, **(I)** seed setting rate, and **(J)** seed length/width ratio. The four ecotypes are: indica inbred (II) and indica hybrid (IH), japonica inbred (JI), and japonica hybrid (JH). Boxplots represent the median (line), 25–75 quartiles (boxes), 5th and 95th percentile values (error bars) and outliers (dot). Different letters above a column indicate a significant difference (*P* < 0.05).

On average, the japonica ecotypes had significantly greater GY and longer GP than indica ecotypes (Figure 1A,E), but they had significantly shorter PL and smaller LW (Figure 1I,J). IH generally had higher GY, FGN, TGW, GP, PH, GPP, PL, lower PN, SS, and LW than II (Figure 1). Similar patterns were found for japonica ecotypes, except the TGW and LW were lower in the hybrid than inbred ecotype (Figure 1).

### The Relationship Between Grain Yield and Other Agronomic Traits

**FIGURE 2:**
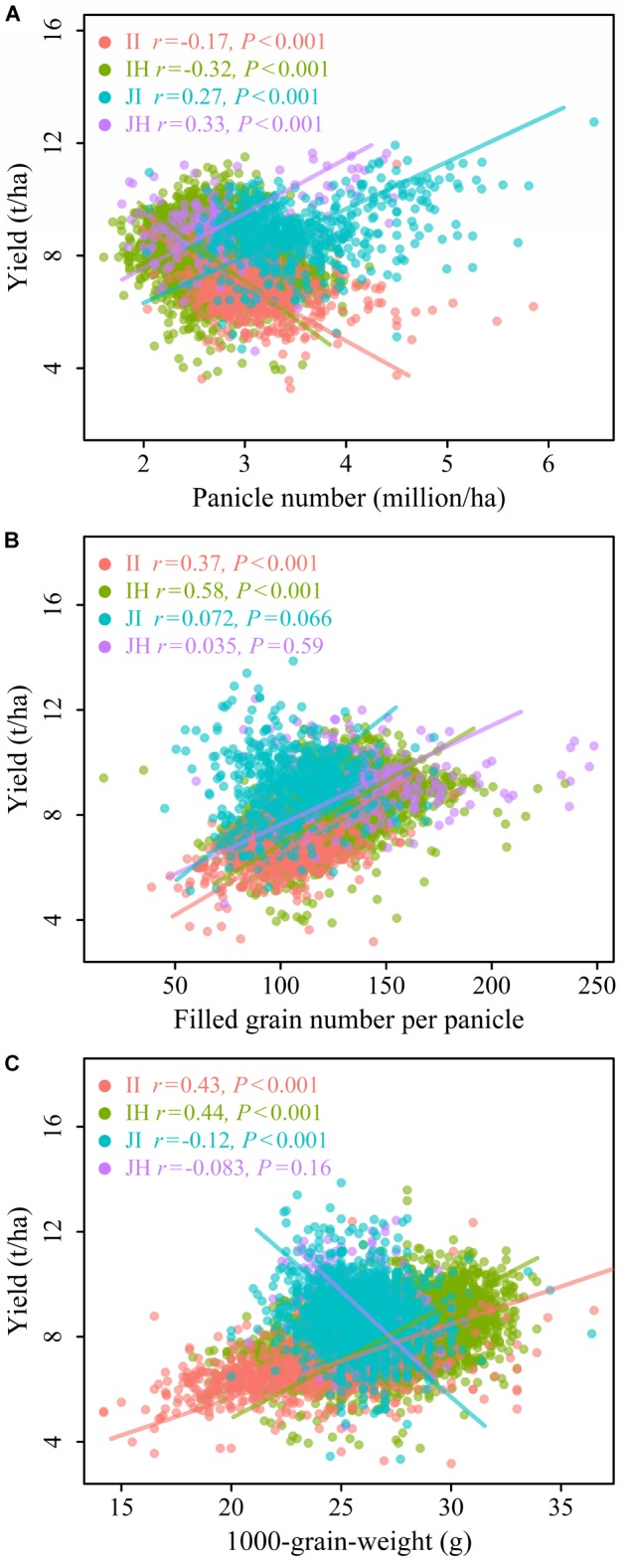
Relationships between grain yield and **(A)** panicle number per unit area, **(B)** filled grain number per panicle, and **(C)** 1,000-grain-weight for four rice ecotypes. The four ecotypes are: indica inbred (II), and indica hybrid (IH), japonica inbred (JI), and japonica hybrid (JH). Different rice ecotypes are represented with different colors. Colored lines present regression plots of different rice ecotypes. The details of these relations using reduced major axis (RMA) regressions are presented in Table 1. Pearson correlation coefficients (*r*) and their significance level (*P*) are shown.

For the relationship between GY and PN (GY-PN), a negative association was found for the II (*r* = -0.17, *P* < 0.001) and IH (*r* = -0.32, *P* < 0.001), but a positive association was found for the JI (*r* = 0.27, *P* < 0.001) and JH (*r* = 0.33, *P* < 0.001; Figure 2A). The slopes of the GY-PN relationships differed significantly among II, IH and JI, while the slope for JH only differed significantly from IH, and not for II and JI (SMA, Table 1 and Figure 2A). Strong positive associations were found between FGN and GY (GY-FGN) for indica ecotypes, but no significant relationships were found within japonica ecotypes (Figure 2B). The GY-FGN relationships differed significantly among the slopes comparing the four ecotypes, except for II and IH (Table 1 and Figure 2B). A positive correlation was found between TGW and GY (GY-TGW) for indica rice (Figure 2C). TGW was found to correlate negatively with yield for the JI rice, and there was no significant correlation with the JH (Figure 2C). The GY-TGW relationships differed in slope significantly among the four rice ecotypes, except for the JI and JH (Table 1 and Figure 2C).

**Table 1:** Standardized major axis (SMA) slopes fitted within individual ecotypes, corresponding to Figure 2, 3.

Figure	Traits	Ecotype	*n*	*R*^2^	*P*	Slope	Low CI	High CI	Pairwise comparison
2A	GY-PN	II	585	0.028	**<0.001**	–1.99	–2.16	–1.84	b
		IH	3965	0.10	**<0.001**	–2.68	–2.75	–2.6	c
		JI	603	0.072	**<0.001**	1.67	1.55	1.81	a
		JH	186	0.11	**<0.001**	1.94	1.69	2.23	ab
2B	GY-FGN	II	592	0.14	**<0.001**	0.047	0.044	0.051	b
		IH	3879	0.34	**<0.001**	0.048	0.047	0.049	b
		JI	649	0.0052	0.066	0.063	0.059	0.068	a
		JH	233	0.0012	0.59	0.038	0.033	0.043	c
2C	GY-TGW	II	717	0.19	**<0.001**	0.28	0.27	0.3	b
		IH	4725	0.20	**<0.001**	0.44	0.43	0.45	a
		JI	1755	0.015	**<0.001**	–0.72	–0.75	–0.69	c
		JH	282	0.007	0.16	–0.78	–0.88	–0.7	c
3A	GY-GP	II	747	0.083	**<0.001**	0.07	0.065	0.075	b
		IH	4766	0.42	**<0.001**	0.068	0.067	0.07	b
		JI	1453	0.067	**<0.001**	0.08	0.076	0.084	a
		JH	300	0.35	**<0.001**	0.091	0.083	0.1	a
3B	GY-PH	II	701	0.05	**<0.001**	0.09	0.084	0.097	b
		IH	4683	0.18	**<0.001**	0.099	0.097	0.1	b
		JI	1717	0.015	**<0.001**	0.13	0.12	0.14	a
		JH	275	0.00085	0.63	0.12	0.11	0.13	a
3C	GY-PL	II	470	0.027	**<0.001**	0.49	0.45	0.54	ab
		IH	3010	0.18	**<0.001**	0.55	0.53	0.57	a
		JI	1322	0.0068	**0.0027**	–0.47	–0.5	–0.45	b
		JH	197	0	0.97	0.5	0.44	0.58	ab
3D	GY-GPP	II	543	0.09	**<0.001**	0.035	0.033	0.038	b
		IH	3403	0.33	**<0.001**	0.037	0.036	0.038	b
		JI	343	0.0094	0.074	0.051	0.046	0.057	a
		JH	160	0.014	0.13	0.026	0.023	0.031	c
3E	GY-SS	II	694	0.019	**<0.001**	0.22	0.2	0.24	b
		IH	4570	0.034	**<0.001**	0.25	0.25	0.26	a
		JI	1115	0.00024	0.60	–0.22	–0.24	–0.21	b
		JH	247	0.0027	0.42	0.21	0.19	0.24	b
3F	GY-LW	II	421	0.056	**<0.001**	–1.88	–2.06	–1.71	b
		IH	3781	0.017	**<0.001**	–3.33	–3.44	–3.23	c
		JI	760	0.0002	0.70	5.5	5.12	5.91	a
		JH	152	0.018	0.095	–3.83	–4.49	–3.26	c

*Data shown is number of observations (n), SMA R^*2*^, P-values and slope (95% CI). Significant differences (P < 0.05) in slopes among ecotypes are represented by the lack of shared letters in the pairwise comparison column. Bold values indicate significant correlations at the level of 0.05.*

*GY, grain yield; PN, panicle number per unit area; FGN, filled grain number per panicle; TGW, 1,000-grain-weight; GP, growth period; PH, plant height; PL, panicle length; GPP, grains per panicle; SS, seed setting rate; and LW, seed length/width ratio. Four rice ecotypes: II, indica inbred; IH, indica hybrid; JI, japonica inbred; and JH, japonica hybrid*.

**FIGURE 3:**
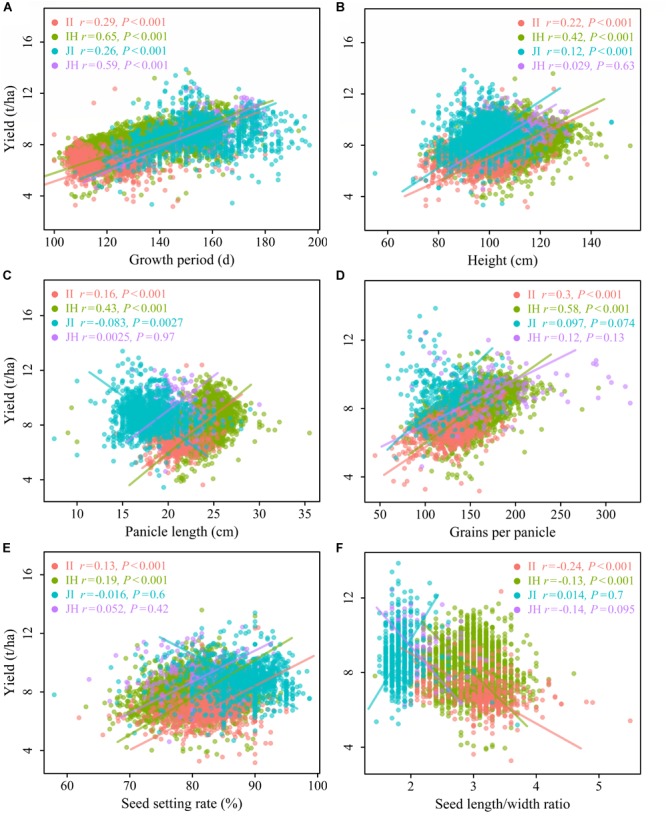
Relationships between grain yield and **(A)** growth period, **(B)** plant height, **(C)** panicle length, **(D)** grains per panicle, **(E)** seed setting rate, and **(F)** seed length/width ratio for the four rice ecotypes. Figure explanations are provided in Figure 2.

GY was significantly and positively correlated with GP for all rice types (GY-GP), which indicates that varieties with a longer life span are associated with higher yield (Figure 3A). PH was found to positively associate with GY for II and IH ecotypes (GY-PH), whilst there was a weak association for JI rice (*r* = 0.12, *P* < 0.001) and a non-significant correlation for JH rice (Figure 3B). The GY-GP and GY-PH relationships differed significantly between indica and japonica ecotypes in their slopes, but the slopes were similar within indica and japonica ecotypes (Table 1 and Figure 3A,B).

There was no significant relationship between GY and PL for japonica ecotypes, whereas there were significant positive associations for II (*r* = 0.16, *P* < 0.001) and IH ecotypes (*r* = 0.43, *P* < 0.001) (Figure 3C). A similar pattern was observed between GY and GPP (GY-GPP) (Figure 3D). The GY-GPP relationships differed significantly among the four rice ecotypes, but not between II and IH (Table 1 and Figure 3D). The SS was found to be associated slightly with GY for indica ecotypes, while non-significant relationships were found in japonica ecotypes (Figure 3E). No significant relationships between LW and GY were found for japonica, but significant negative relationships were found for both indica ecotypes (Figure 3F).

### Direct Effects of Agronomic Traits on Grain Yield

**FIGURE 4:**
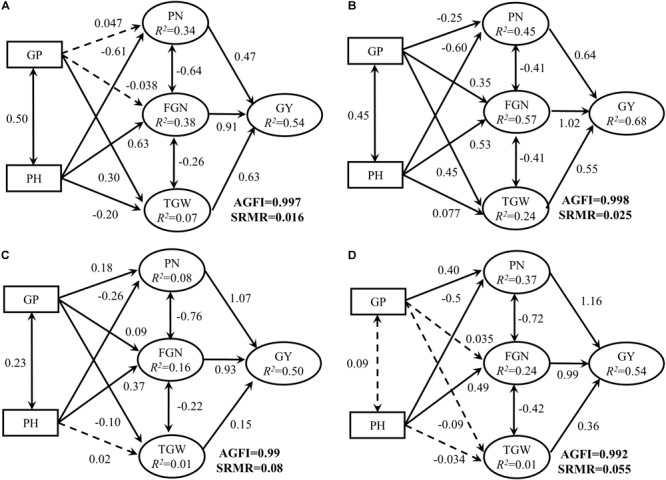
Structural equation models fitted to connections among panicle number per unit area (PN), filled grain number per panicle (FGN), 1000-grain-weight (TGW), and yield (GY) and the effects of growth period (GP) and plant height (PH) on PN, FGN, and TGW in **(A)** indica inbred (II), **(B)** indica hybrid (IH), **(C)** japonica inbred, and **(D)** japonica hybrid (JH). Numbers adjacent to arrows represent the standardized partial regression coefficients (β). *R*^2^ indicates the proportion of variance explained. Solid arrows represent significant paths (*P* < 0.05), and dashed arrows represent non-significant paths (*P* > 0.05). Adjusted goodness-of-fit statistic (AGFI) and standardized root mean square residual (SRMR) were shown in the bottom right corner. Relative effect sizes were presented in Table 2.

The models adequately fit the data, based on adjusted goodness-of-fit statistic (AGFI, with all AGFI values > 0.99) and standardized root mean square residual (SRMR, with all SRMR values < 0.08). Considering only the direct effects, yield was strongly influenced in all rice ecotypes by PN, FGN and TGW (Table 2 and Figure 4).

**Table 2:** Pathways and standardized partial regression coefficients across four rice ecotypes and proposed interpretations based on structural equation models (SEMs).

Effect	II	IH	JI	JH	Proposed interpretation
PN → GY	0.47	0.64	1.07	1.16	Increasing PN contributes to higher GY.
FGN → GY	0.91	1.02	0.93	0.99	GY variation is strongly influenced by FGN.
TGW → GY	0.63	0.55	0.15	0.36	GY increases with increasing TGW.
GP → PN	NS	–0.25	0.18	0.40	GP either increases or decreases PN depending on rice ecotypes.
GP → FGN	NS	0.35	0.09	NS	GP regulates FGN.
GP → TGW	0.30	0.45	–0.10	NS	GP alters TGW.
PH → PN	–0.61	–0.60	–0.26	–0.50	PN is strongly regulated by PH.
PH → FGN	0.63	0.53	0.37	0.49	PH controls FGN.
PH → TGW	–0.20	0.077	NS	NS	PH either increases or decreases TGW depending on rice ecotypes.
GP → GY	0.18	0.44	0.26	0.47	Indirect effect of GP on GY.
PH → GY	0.16	0.20	0.069	–0.11	Indirect effect of PH on GY.

*Four rice ecotypes: indica inbred (II, 767 varieties), and indica hybrid (IH, 4814 varieties), japonica inbred (JI, 1809 varieties) and japonica hybrid (JH, 296 varieties). Magnitude of the pathway is presented by standardized partial regression coefficients fitted by the SEM. All significant pathways are shown (P < 0.05). NS means no significant effect (P > 0.05). Abbreviations are provided in Table 1*.

For II ecotype, the FGN, TGW and PN accounted for 54% (*R*^2^ = 0.54) of the variation in GY. FGN had the strongest positive effects on GY (β = 0.91, *P* < 0.05, Table 2 and Figure 4A), which means if FGN was increased by one standard deviation while TGW and PN were held constant, GY would be expected to increase by 0.91 standard deviations. PN and TGW positively affected the GY with β = 0.63 and 0.47 (*P* < 0.05, Table 2 and Figure 4A), respectively.

For IH ecotype, the FGN, TGW, and PN accounted for 68% (*R*^2^ = 0.68) of the variation in GY (Table 2 and Figure 4B). FGN had the strongest positive effects on GY (β = 1.02, *P* < 0.05), followed by PN and TGW with β = 0.64 and 0.55 (*P* < 0.05).

For JI ecotype, the FGN, TGW, and PN accounted for 50% (*R*^2^ = 0.50) of the variation in GY (Table 2 and Figure 4C). PN had the strongest positive effects on GY (β = 1.07, *P* < 0.05), followed by FGN and TGW with β = 0.93 and 0.15 (*P* < 0.05).

For JH ecotype, the FGN, TGW, and PN accounted for 54% (*R*^2^ = 0.54) of the variation in GY (Table 2 and Figure 4D). PN had the strongest positive effects on GY (β = 1.16, *P* < 0.05), followed by FGN and TGW with β = 0.99 and 0.36 (*P* < 0.05).

### Indirect Effects of Growth Period and Plant Height on Grain Yield

Positive effects of GP on GY were consistently observed in each ecotype via direct yield components (Table 2 and Figure 4). For the II ecotype, the indirect effects of GP via PN and FGN were non-significant. However, the positive effect via TGW was found to be significant (Table 2 and Figure 4A). For the IH ecotype, the indirect effect of GP via PN was negative, with GP having a positive effect via the FGN and TGW (Table 2 and Figure 4B). In JI ecotype, the indirect effects of GP via FGN and TGW were weak in contrast to the positive effects via PN (Table 2 and Figure 4C). In JH ecotype, a significant indirect effect was found through PN only (Table 2 and Figure 4D).

Positive effects of PH on GY were found for indica ecotypes. However, the effect on the JI was not prominent, and a negative effect was found for the JH (Table 2 and Figure 4). In all rice ecotypes, PH strongly and negatively affected GY through PN, and there were strong positive effects on GY through FGN (Table 2 and Figure 4). PH also indirectly affected GY via TGW, with significant negative effects found for the II (β = -0.20, *P* < 0.05), and significant positive effects found for the IH (β = 0.077, *P* < 0.05). However, there were no significant effects on the JH (β = -0.034, *P* > 0.05) or JI (β = 0.02, *P* > 0.05, Table 2 and Figure 4).

## Discussion

Our findings from these large datasets confirmed that agronomic characters of rice play a crucial role in determining yield, which aligns with a number of previous findings (Pb Samonte et al., 1998; Sheehy et al., 2001; Casanova et al., 2002; Fageria et al., 2011; Qian et al., 2016; Zeng et al., 2017; Zhang Y. et al., 2017). Our results clearly showed that associations between agronomic traits and yield were ecotype-dependent. For both indica ecotypes, we found high values of FGN, TGW, PH, PL, GPP and SS, and low values of PN, LW and long GP account for high GY (Figure 2, 3). For both japonica ecotypes, varieties with high PN and long GP have high yield (Figure 2, 3).

### The Correlations Between Panicle Number per Unit Area and Grain Yield for Different Rice Ecotypes

PN plays a critical role in determining GY and it was found that this main yield component is greatly affected by both environmental conditions and management (Garcia et al., 2015). Yield enhancement via improvement of crop management is generally achieved by increasing the panicle number (Sadras, 2007; Wang et al., 2017). PN is determined by the number of seedlings and tillers produced per seedling. Transplanted rice generally produce more tillers than directly sown rice. In our study, associations of GY and PN showed opposite patterns in indica ecotypes (negatively correlated, Figure 2A) and japonica ecotypes (positively correlated, Figure 2A). The PN increased significantly as the density of plants within a given unit of area increased, but there was a strong trade-off between FGN and PN (Figure 4). This dynamic compensation of FGN appears to limit potential GY benefits as the panicle number increases, especially for indica ecotypes (Figure 2). Additionally, when planting rates are very high, tillers may not develop well, or may even die before they can produce a panicle. This would lead to decreased PN. Other field management practices, such as nitrogen application, or weed, pests and diseases management may also influence PN. However, for japonica rice, rice varieties that have higher tillering rates and planting in high density might be still appropriate choices.

### The Correlations Between Filled Grain Number per Panicle and Grain Yield for Different Rice Ecotypes

FGN a multiplication product of GPP and SS. The number of GPP is mainly determined by the panicle architecture, including PL and the number and length of primary, secondary and higher order branches (Sakamoto and Matsuoka, 2008; Kovi et al., 2011a). Architecture is also one of the main determinants for all gramineous crop yield (Furutani et al., 2006; Qi et al., 2011). SS is often responsible for determining grain number, and is largely affected by external environment. Several genes related to SS have been reported for rice, such as *PTB1* which controls pollen tube growth, *OsSPX1* which is responsible for semi-male sterility and *OsLAC13* which affects hydrogen peroxide dynamics and mitochondrial integrity (Li et al., 2013; Zhang et al., 2016; Yu et al., 2017).

To further increase yield and to break the yield stagnation, breeding efforts have managed to expand the yield sink capacity, which is the maximum size of sink organs to be harvested, by increasing the GPP. As a result, cultivars with large panicles and/or numerous spikelets per panicle have become available, such as the new plant type of the International Rice Research Institute (Peng et al., 1999), ‘super’ hybrid rice in China. These cultivars, however, frequently lack high yield potential due to poor SS and slow grain-filling rate (Yang and Zhang, 2010). Moreover, SS is susceptible to the prevailing environmental conditions. Generally, the higher the temperature, the shorter the duration of the grain-filling period and the faster the grain-filling rate (Fujita et al., 1984; Yang and Zhang, 2010; Das et al., 2018). It was found that during the mid and late grain-filling stages, a controlled or moderate soil drying that allows the plant to rehydrate overnight and doesn’t severely inhibit photosynthesis can greatly promote whole-plant senescence and assimilate remobilization (Yang and Zhang, 2010). Therefore, it is necessary to integrate breeding approaches with crop management to improve FGN, especially for indica rice.

### The Correlations Between 1000-Grain-Weight and Grain Yield for Different Rice Ecotypes

The quantitative trait of TGW is determined by both grain size and grain filling rate, which is characterized by the three dimensions of grain length, width and height (Huang et al., 2013; Xie et al., 2015). Grain weight is mainly governed by genetic factors, whereas grain filling rate is affected by external environmental conditions. In this study, we found that the TGW influences yield greatly in both indica ecotypes, but neither japonica ecotype (Figure 2C). The average TGW is the same for the JI and JH (25.8 g), but differs for the II (24.0 g) and IH (27.1 g). Apparently TGW does not account for the yield advantages of japonica. The LW correlates negatively with grain weight, though the degree of correlation varied (Xing and Zhang, 2010; Song et al., 2015). Additionally, the LW showed a significant negative correlation with the yield of indica rice (Figure 3F). However, for indica ecotypes with high LW, a greater width may contribute to a higher TGW and thus a higher yield. These results are consistent with the opinion that the grain length and width are important factors influencing the yield (Zhang et al., 2012; Si et al., 2016). Compared with other yield traits, TGW was consistent across different environments, so it could be a potential candidate for yield improvement, especially for indica ecotypes (Yang and Zhang, 2010). It is worth noting that an increased TGW may lead to a decrease in FGN and a decreased yield overall. The trade-off between TGW and the number of FGN should be carefully considered in rice breeding (Yang and Zhang, 2010).

### The Correlations Between Growth Period and Grain Yield for Different Rice Ecotypes

In addition to the traits mentioned above, other traits, such as GP and PH also contribute to the final GY (Sakamoto and Matsuoka, 2008). Although these traits do not directly affect GY, they may influence GY indirectly through the direct component traits such as PN, FGN and TGW. For all rice ecotypes, the associations between GP and GY were found to be much stronger than between others (Table 1), even stronger than among some direct component traits.

The GP differs greatly and depends upon the genetic characteristics and the prevailing environmental conditions (Kawano and Tanaka, 1968; Peng et al., 2004). Rice varieties with longer GP have more time to absorb sunlight, water and nutrients which may increase spikelets, panicles and sufficient leaf area (Vergara et al., 1964; Craufurd and Wheeler, 2009). It was reported that the grain-filling period is longer in japonica than indica ecotypes, even under controlled environmental conditions (Fujita et al., 1984). In the present study, we found that GP was significantly longer in japonica ecotypes than indica (152.59 and 130.89 d, respectively, Figure 1E), which may be responsible for the relatively higher GY in japonica (8.62 and 7.81 t/ha for japonica and indica ecotypes, respectively, Figure 1A). Because the GP are normally limited in several regions, breeders should pay more attention to relatively long-lasting rice cultivars. For farmers, proper seeding and harvesting time needs to be considered in the pursuit of larger yields. Additionally, future crop productivity will depend on the extent of change in GP under the changing climatic scenario (Mall and Aggarwal, 2002).

### The Correlations Between Plant Height and Grain Yield for Different Rice Ecotypes

To achieve high yields, an optimal architecture, often determined by PH, is necessary (Xue et al., 2008). Closely related to biomass production, PH is an important morphological trait affecting yield performance. Both molecular cloning and functional analyses of several genes associated with PH in rice have illuminated interconnections to the synthesis and regulation of the phytohormone, i.e., gibberellin (Ikeda, 2001; Matusmoto et al., 2016). The introduction of semi-dwarf cultivars improves the harvest index and increases the GY by enhancing lodging resistance (Spielmeyer et al., 2002; Kovi et al., 2011b). However, if the plants are too short, the yield potential will be negatively affected. Based on their experience in super-hybrid rice breeding, Yuan (2017) suggested that increasing PH is an effective way to increase GY. Therefore, in the absence of lodging, it is essential to increase PH in order to increase yield (Zhang Y. et al., 2017). PH was found to be higher in hybrid than in inbred ecotypes for both japonica (98.74 cm for inbred and 108.61 cm for hybrid rice) and indica (96.45 cm for inbred and 108.70 cm for hybrid rice), which may be one reason that leads to the higher yield of hybrid rice varieties. It should be noted that associations between PH and yield depend on rice ecotypes. PH had the strongest positive effect on GY for the IH (Tables 1, 2 and Figure 3B). Thus, breeders focusing on IH ecotype may consider to increase the PH to improve yield potential.

### Grain Yield Improvement in the Past and Future

In this study, we found that the year of release significantly correlated with agronomic traits, especially for PN of indica inbred, PL of indica inbred, FGN, GPP, PH, and GY of all ecotypes (Supplementary Figures S1–S10). This trend of agronomic traits over time reflects breeder selection. Further, the year of release did not affect the simple correlation coefficient, as shown in Figure 2, 3 (partial correlation with year of release as partial variable, Supplementary Table S2), which means that the progress achieved in rice breeding during the past does not change the associations between GY and other agronomic traits.

Direct and indirect yield components compensate with each other, and an increase or decrease in one component does not necessarily result in an overall increase in GY. It is difficult to increase rice yield potential by improving a single yield trait (Cassman, 1994; Yang and Zhang, 2010; Huang et al., 2011; Li et al., 2015; Qian et al., 2016; Zeng et al., 2017). For example, an increase in GY not only needs to enlarge sink size by increasing the number of panicles, but also requires adjustment of other yield formation processes. Additionally, many studies have found that the degree of heterosis in inter-subspecies indica-japonica hybrids is larger than that of intra-subspecies hybrids (Yuan, 2017). For this reason, crosses between indica and japonica subspecies for the development of super hybrid rice have been introduced gradually since the 1980s. However, there are many challenges in developing such heterosis in breeding programs because the inharmonious genetic backgrounds of indica and japonica cause sterility in the F1 generation (Qian et al., 2016). This problem has been addressed with the discovery and molecular characterization of wide compatibility genes (Chen et al., 2008; Zhu et al., 2017). Thus, breeding super high-yielding rice likely involves combining favorable agronomic traits with strong heterosis via indica-japonica hybridization in the future.

Grain yield can be increased by breeding new rice varieties with greater yield potential, but also by improving crop and resource management. Crop management includes nutrient and water management, planting methods and density, and has proven to be highly effective in improving rice GY (Cassman, 1994; Tao et al., 2006; Usui et al., 2016; Kimura and Shimono, 2017; Das et al., 2018). By adopting and/or practicing proper crop management techniques, yield components like PN, FGN, SS, GP and PH can be improved to get better rice yields (Yang and Zhang, 2010; Wang et al., 2017).

## Conclusion

The present study made use of the trait data of four rice ecotypes released in China from 1978 to 2017. We found that correlations between agronomic traits and yield depends on the ecotypes of rice variety. For both II and IH ecotypes, improvement in GY should focus on increasing the FGN, TGW, GP, PH, PL, GPP, SS, decreasing PN and LW. In JI and JH ecotypes, GY can be improved by increasing PN and GP. These results may be utilized in breeding and planting different rice ecotypes.

## Author Contributions

JZ, RL, and ML conceived the research. RL, ML, and SL collected and analyzed data. ML, RL, UA, and JZ wrote the manuscript.

## Conflict of Interest Statement

The authors declare that the research was conducted in the absence of any commercial or financial relationships that could be construed as a potential conflict of interest.

## Footnotes

1 http://faostat.fao.org/

2 http://data.stats.gov.cn/

3 http://www.ricedata.cn/variety/

4 http://www.ricedata.cn/variety/

5 http://www.cgris.net/

## Abbreviations

FGN: filled grain number per panicle
GP: growth period
GPP: grains per panicle
GY: grain yield
IH: indica hybrid
II: indica inbred
JH: japonica hybrid
JI: japonica inbred
LW: seed length/width ratio
PH: plant height
PL: panicle length
PN: panicle number per unit area
SS: seed setting rate
TGW: 1000-grain-weight
