# Supplementary material for: Exploring the Relationships Between Yield and Yield-Related Traits for Rice Varieties Released in China From 1978 to 2017
Source: Front Plant Sci. 2019 May 7;10:543. doi: 10.3389/fpls.2019.00543 (PMC6514245; doi:10.3389/fpls.2019.00543)
Supplement: Supplementary file 1 [file Table_1.docx]

**Supplementary Table S1 This file contains the computer code that accompanies the paper.**

**Note: Before you run the code, please delete the first row of Data Sheet 1**

##################################################.

**Codes for Table 1**

##################################################

install.packages("smatr")#####install packages

library(smatr)#####load package

rice<-read.csv("Data sheet 1.csv")##### load data

str(rice)##### Compactly display the structure of data

names(rice) <- c("var","ecotype","prov","year","y","gp","ph","pl","pn","ss","tgw","gpp","lw","fgn")#####rename column

SMA<-sma(y~pn*ecotype,data=rice,multcomp=TRUE,multcompmethod="adjusted")#####sma analysis for GY-PN

summary(SMA)#####summary for output

multcompmatrix(SMA,sort=TRUE)######compare slopes for different ecotype

plot(SMA)#####plot sma regression

SMA<-sma(y~fgn*ecotype,data=rice,multcomp=TRUE,multcompmethod="adjusted")#####sma analysis for GY- FGN

summary(SMA)

multcompmatrix(SMA,sort=TRUE)

plot(SMA)

SMA<-sma(y~tgw*ecotype,data=rice,multcomp=TRUE,multcompmethod="adjusted")#####sma analysis for GY- TGW

summary(SMA)

multcompmatrix(SMA,sort=TRUE)

plot(SMA)

SMA<-sma(y~gp*ecotype,data=rice,multcomp=TRUE,multcompmethod="adjusted")#####sma analysis for GY- GP

summary(SMA)

multcompmatrix(SMA,sort=TRUE)

plot(SMA)

SMA<-sma(y~ph*ecotype,data=rice,multcomp=TRUE,multcompmethod="adjusted")#####sma analysis for GY- PH

summary(SMA)

multcompmatrix(SMA,sort=TRUE)

plot(SMA)

SMA<-sma(y~pl*ecotype,data=rice,multcomp=TRUE,multcompmethod="adjusted")#####sma analysis for GY- PL

summary(SMA)

multcompmatrix(SMA,sort=TRUE)

plot(SMA)

SMA<-sma(y~gpp*ecotype,data=rice,multcomp=TRUE,multcompmethod="adjusted")#####sma analysis for GY- GPP

summary(SMA)

multcompmatrix(SMA,sort=TRUE)

plot(SMA)

SMA<-sma(y~ss*ecotype,data=rice,multcomp=TRUE,multcompmethod="adjusted")#####sma analysis for GY- SS

summary(SMA)

multcompmatrix(SMA,sort=TRUE)

plot(SMA)

SMA<-sma(y~lw*ecotype,data=rice,multcomp=TRUE,multcompmethod="adjusted")#####sma analysis for GY- LW

summary(SMA)

multcompmatrix(SMA,sort=TRUE)

plot(SMA)

###########################################################

**Codes for Figure 2.**

**Codes for figure 3 is similar to Figure 2, thus we do not shown here.**

###########################################################

library(extrafont) #####load package

library(smatr) #####load package

library(extrafont) #####load package

library(grid) #####load package

library(gridExtra) #####load package

library(scales) #####load package

library(Cairo) #####load package

font_import()#####import font

loadfonts(device="win") #####load font

rice<-read.csv("Data sheet 1.csv")#####load data

names(rice) <- c("var","ecotype","prov","year","y","gp","ph","pl","pn","ss","tgw","gpp","lw","fgn")#####rename column

SMApn<-sma(y~pn*ecotype,data=rice,multcomp=TRUE,multcompmethod="adjusted")#####sma analysis for GY- PN

SMAfgn<-sma(y~fgn*ecotype,data=rice,multcomp=TRUE,multcompmethod="adjusted")#####sma analysis for GY- FGN

SMAtgw<-sma(y~tgw*ecotype,data=rice,multcomp=TRUE,multcompmethod="adjusted")#####sma analysis for GY- TGW

png("figure2.png",width = 6,height = 15,units= "in",res= 1200,pointsize = 4) #####Plot figure 2

par( mar=c(6,6,1,1))

par(mgp = c(4,1.5,0))

par(mfrow=c(3,1))

par(col=1)

par(font=2)

par(family="Times New Roman")

par(cex=2)

par(cex.lab=2.4)

par(cex.axis=2.2)

par(lwd=2)

cols <-c("#7CAE00","#F8766D","#C77CFF","#00BFC4")

plot(SMApn, lwd=4,col = alpha(cols, 0.6),xlab="Panicle number (million/ha)",ylab="Yield (t/ha)",pch=1,ylim=c(2,16), axes = FALSE)

axis(side=1,lwd=2)

axis(side=2,lwd=2,at=c(4,8,12,16))

par(family="Times New Roman")

legend("topleft", legend = c(as.expression(bquote(II~italic(" r"*"="*.(-0.17)*","*" P"*"<"*.(0.001)))),

as.expression(bquote(IH~italic("r"*"="*.(-0.32)*","*" P"*"<"*.(0.001)))),

as.expression(bquote(JI~italic(" r"*"="*.(0.27)*","*" P"*"<"*.(0.001)))),

as.expression(bquote(JH~italic("r"*"="*.(0.33)*","*" P"*"<"*.(0.001))))),col = c("#F8766D","#7CAE00","#00BFC4","#C77CFF"),

text.col=c("#F8766D","#7CAE00","#00BFC4","#C77CFF"),cex=2,bty = "n", pch = c(19,19,19,19))

plot(SMAfgn,lwd=4, col = alpha(cols, 0.6),xlab="Filled grain number per panicle",ylab="Yield (t/ha)",pch=1,ylim=c(3,18), axes = FALSE)

axis(side=1,lwd=2)

axis(side=2,lwd=2,at=c(4,8,12,16))

legend("topleft", legend = c(as.expression(bquote(II~italic(" r"*"="*.(0.37)*","*" P"*"<"*.(0.001)))),

as.expression(bquote(IH~italic("r"*"="*.(0.58)*","*" P"*"<"*.(0.001)))),

as.expression(bquote(JI~italic(" r"*"="*.(0.072)*","*" P"*"="*.(0.066)))),

as.expression(bquote(JH~italic("r"*"="*.(0.035)*","*" P"*"="*.(0.59))))),col = c("#F8766D","#7CAE00","#00BFC4","#C77CFF"),

text.col=c("#F8766D","#7CAE00","#00BFC4","#C77CFF"),cex=2,bty = "n", pch = c(19,19,19,19))

plot(SMAtgw,lwd=4, col = alpha(cols, 0.6),xlab="1000-grain-weight (g)",ylab="Yield (t/ha)",pch=1,ylim=c(3,18), axes = FALSE)

axis(side=1,lwd=2)

axis(side=2,lwd=2,at=c(4,8,12,16))

legend("topleft", legend = c(as.expression(bquote(II~italic(" r"*"="*.(0.43)*","*" P"*"<"*.(0.001)))),

as.expression(bquote(IH~italic("r"*"="*.(0.44)*","*" P"*"<"*.(0.001)))),

as.expression(bquote(JI~italic(" r"*"="*.(-0.12)*","*" P"*"<"*.(0.001)))),

as.expression(bquote(JH~italic("r"*"="*.(-0.083)*","*" P"*"="*.(0.16))))),col = c("#F8766D","#7CAE00","#00BFC4","#C77CFF"),

text.col=c("#F8766D","#7CAE00","#00BFC4","#C77CFF"),cex=2,bty = "n", pch = c(19,19,19,19))

dev.off()

###########################################################

**Codes for Figure 4 and Table 2. SEM**

###########################################################

library(lavaan)

rice<-read.csv("Data sheet 1.csv")#####load data

names(rice) <- c("var","ecotype","prov","year","y","gp","ph","pl","pn","ss","tgw","gpp","lw","fgn")#####rename column

##########sem for Indica inbred

data<-subset(rice,ecotype=='Indica inbred')

sitemod <- 'y~pn+fgn+tgw

pn~~fgn

fgn~~tgw

fgn~gp+ph

pn~gp+ph

tgw~gp+ph

gp~~ph' ####################model

sitemod.fit <- sem(sitemod,data=data,missing="ML",fixed.x=FALSE, meanstructure = TRUE) ##############sem anylysis

summary(sitemod.fit,standardized=TRUE) #########summary of the results

fitMeasures(sitemod.fit,c("gfi","srmr"))#########fit measures to assess the global fit of a sem model

###########sem for Indica hybrid

data<-subset(rice,ecotype=='Indica hybrid')

sitemod <- 'y~pn+fgn+tgw

pn~~fgn

fgn~~tgw

fgn~gp+ph

pn~gp+ph

tgw~gp+ph

gp~~ph' ####################model

sitemod.fit <- sem(sitemod,data=data,missing="ML",fixed.x=FALSE,meanstructure = TRUE) ##############sem anylysis

summary(sitemod.fit,standardized=TRUE) #########summary of the results

fitMeasures(sitemod.fit,c("gfi","srmr"))#########fit measures to assess the global fit of a sem model

###########sem for Japonica inbred

data<-subset(rice,ecotype=='Japonica inbred')

sitemod <- 'y~pn+fgn+tgw

pn~~fgn

fgn~~tgw

fgn~gp+ph

pn~gp+ph

tgw~gp+ph

gp~~ph' ####################model

sitemod.fit <- sem(sitemod,data=data,missing="ML",fixed.x=FALSE,meanstructure = TRUE) ##############sem anylysis

summary(sitemod.fit,standardized=TRUE) #########summary of the results

fitMeasures(sitemod.fit,c("gfi","srmr"))#########fit measures to assess the global fit of a sem model

###########sem for Japonica hybrid

data<-subset(rice,ecotype=='Japonica hybrid')

sitemod <- 'y~pn+fgn+tgw

pn~~fgn

fgn~~tgw

fgn~gp+ph

pn~gp+ph

tgw~gp+ph

gp~~ph' ####################model

sitemod.fit <- sem(sitemod,data=data,missing="ML",fixed.x=FALSE,meanstructure = TRUE) ##############sem anylysis

summary(sitemod.fit,standardized=TRUE) #########summary of the results

fitMeasures(sitemod.fit,c("gfi","srmr"))#########fit measures to assess the global fit of a sem model
