# Supplementary material for: Exploring the Relationships Between Yield and Yield-Related Traits for Rice Varieties Released in China From 1978 to 2017
Source: Front Plant Sci. 2019 May 7;10:543. doi: 10.3389/fpls.2019.00543 (PMC6514245; doi:10.3389/fpls.2019.00543)
Supplement: Supplementary file 2 [file Table_2.DOCX]

**Supplement Table S2**

Table S2 Partial correlation coefficients with year of release as partial variable and Pearson correlation coefficients between grain yield and other agronomic traits for different rice ecotypes.

| **Traits** | **Ecotypes** | **n** | **Partial correlation** | ***P*** | **Pearson correlation** | ***P*** |
| --- | --- | --- | --- | --- | --- | --- |
| GY- PN | II | 585 | 0.0055 | 0.89 | -0.17 | <0.001 |
|  | IH | 3965 | -0.28 | <0.001 | -0.32 | <0.001 |
|  | JI | 603 | 0.34 | <0.001 | 0.27 | <0.001 |
|  | JH | 186 | 0.35 | <0.001 | 0.33 | <0.001 |
| GY- FGN | II | 592 | 0.21 | <0.001 | 0.37 | <0.001 |
|  | IH | 3879 | 0.55 | <0.001 | 0.58 | <0.001 |
|  | JI | 649 | -0.082 | 0.038 | 0.072 | 0.066 |
|  | JH | 233 | -0.00092 | 0.99 | 0.035 | 0.59 |
| GY- TGW | II | 717 | 0.51 | <0.001 | 0.43 | <0.001 |
|  | IH | 4725 | 0.45 | <0.001 | 0.44 | <0.001 |
|  | JI | 1755 | -0.088 | <0.001 | -0.12 | <0.001 |
|  | JH | 282 | -0.063 | 0.29 | -0.083 | 0.16 |
| GY- GP | II | 747 | 0.35 | <0.001 | 0.29 | <0.001 |
|  | IH | 4766 | 0.67 | <0.001 | 0.65 | <0.001 |
|  | JI | 1453 | 0.27 | <0.001 | 0.26 | <0.001 |
|  | JH | 290 | 0.58 | <0.001 | 0.59 | <0.001 |
| GY- PH | II | 701 | 0.11 | 0.0031 | 0.22 | <0.001 |
|  | IH | 4683 | 0.40 | <0.001 | 0.42 | <0.001 |
|  | JI | 1717 | 0.04 | 0.096 | 0.12 | <0.001 |
|  | JH | 275 | 0.004 | 0.95 | 0.029 | 0.63 |
| GY- PL | II | 470 | 0.089 | 0.05 | 0.16 | <0.001 |
|  | IH | 3010 | 0.42 | <0.001 | 0.43 | <0.001 |
|  | JI | 1322 | -0.11 | <0.001 | -0.083 | 0.0027 |
|  | JH | 197 | -0.00089 | 0.99 | 0.0025 | 0.97 |
| GY-GPP | II | 543 | 0.092 | 0.032 | 0.3 | <0.001 |
|  | IH | 3403 | 0.54 | <0.001 | 0.58 | <0.001 |
|  | JI | 343 | -0.011 | 0.84 | 0.097 | 0.074 |
|  | JH | 160 | 0.11 | 0.15 | 0.12 | 0.13 |
| GY-SS | II | 694 | 0.19 | <0.001 | 0.13 | <0.001 |
|  | IH | 4570 | 0.18 | <0.001 | 0.19 | <0.001 |
|  | JI | 1115 | 0.0033 | 0.91 | -0.016 | 0.6 |
|  | JH | 247 | 0.059 | 0.36 | 0.052 | 0.42 |
| GY-LW | II | 421 | -0.28 | <0.001 | -0.24 | <0.001 |
|  | IH | 3781 | -0.19 | <0.001 | -0.13 | <0.001 |
|  | JI | 760 | 0.013 | 0.72 | 0.014 | 0.7 |
|  | JH | 152 | -0.14 | 0.078 | -0.14 | 0.095 |

*Note:* Grain yield (GY), panicle number per unit area (PN), filled grain number per panicle (FGN), 1000-grain-weight (TGW), growth period (GP), plant height (PH), panicle length (PL), grains per panicle (GPP), seed setting rate (SS) and seed length/width ratio (LW). Four rice ecotypes: indica inbred (II), and indica hybrid (IH), japonica inbred (JI) and japonica hybrid (JH).
