# Supplementary material for: Exploring the Relationships Between Yield and Yield-Related Traits for Rice Varieties Released in China From 1978 to 2017
Source: Front Plant Sci. 2019 May 7;10:543. doi: 10.3389/fpls.2019.00543 (PMC6514245; doi:10.3389/fpls.2019.00543)
Supplement: Supplementary file 3 [file Presentation_1.pdf]

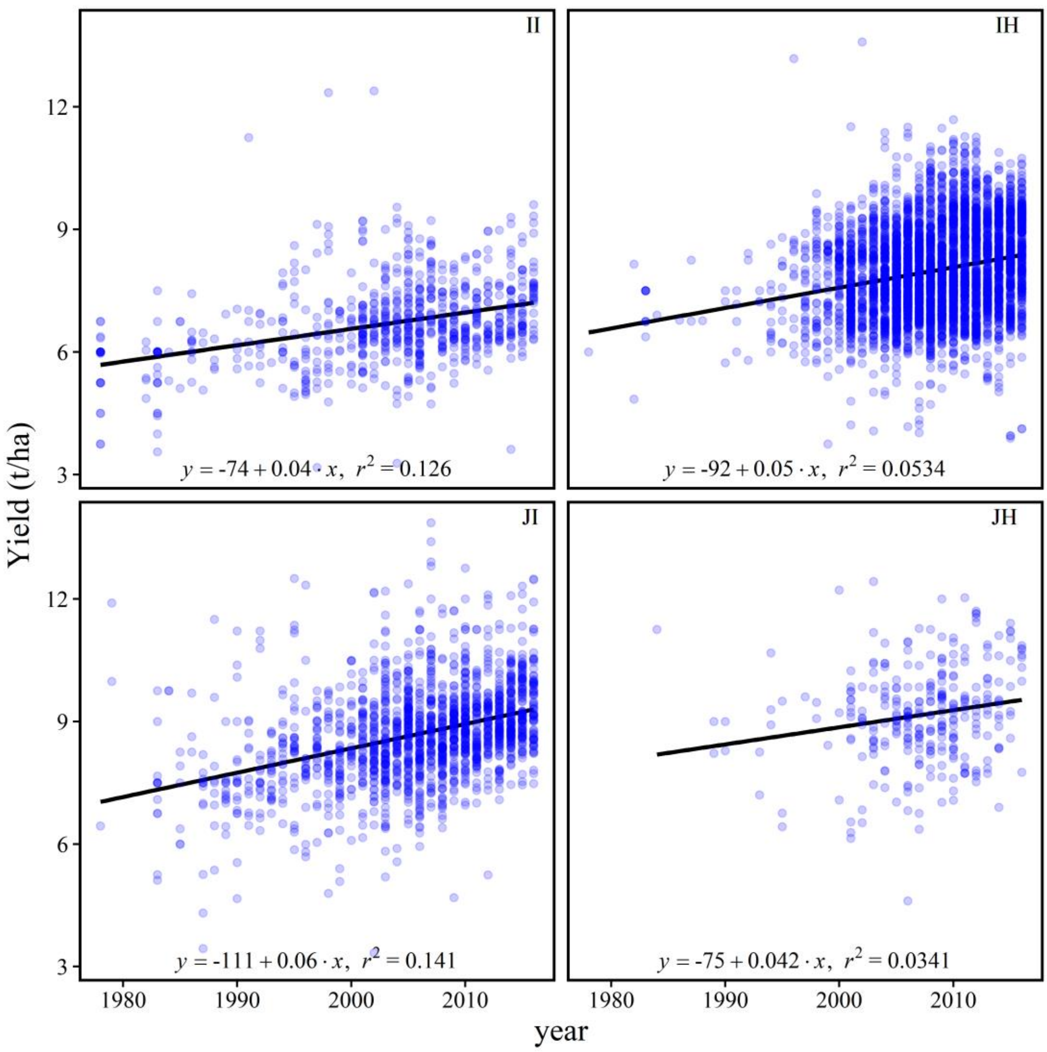

Figure S1. Relationships between year of release and grain yield for different rice ecotypes using least-squares regressions. The four ecotypes are: indica inbred (II), and indica hybrid (IH), japonica inbred (JI) and japonica hybrid (JH).

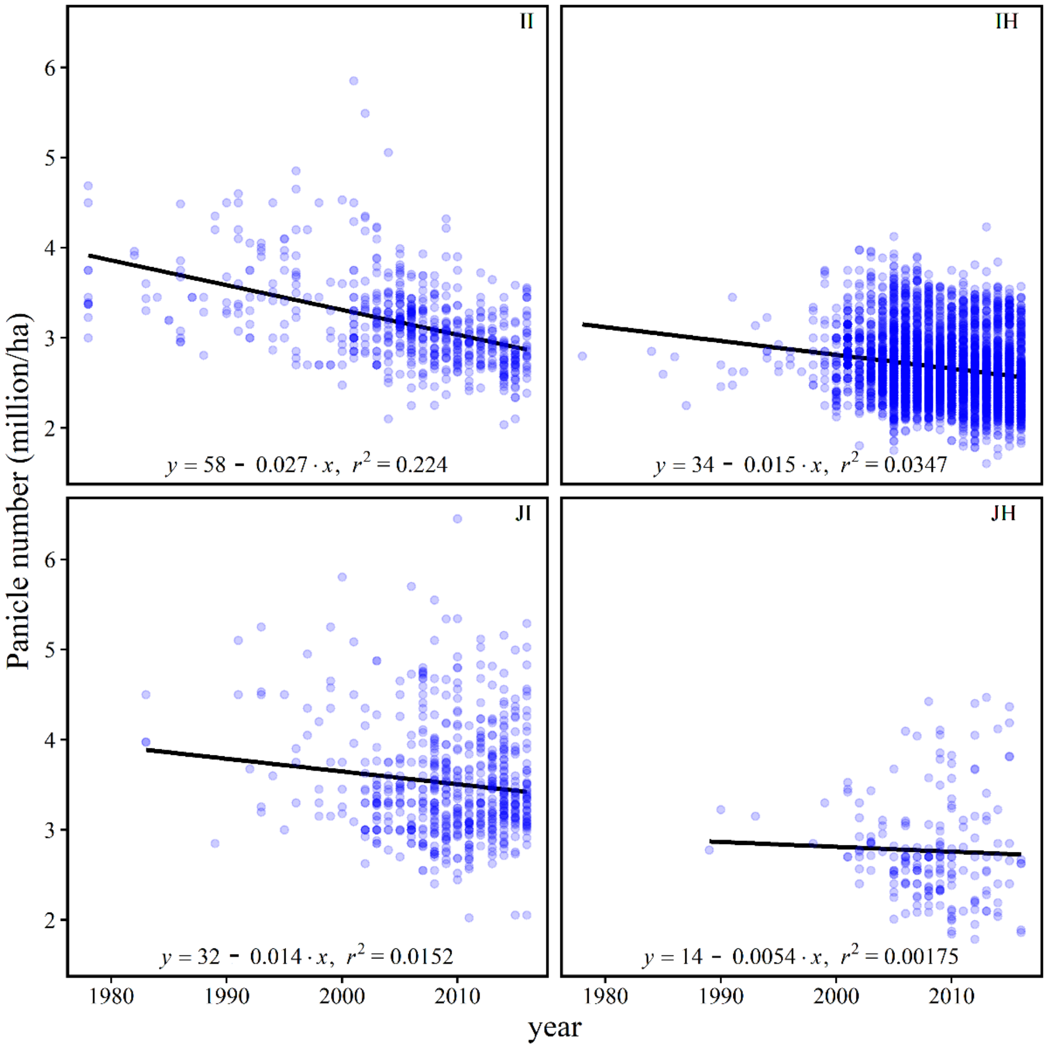

Figure S2. Relationships between year of release and panicle number per unit area for different rice ecotypes using least-squares regressions. The four ecotypes are: indica inbred (II), and indica hybrid (IH), japonica inbred (JI) and japonica hybrid (JH).

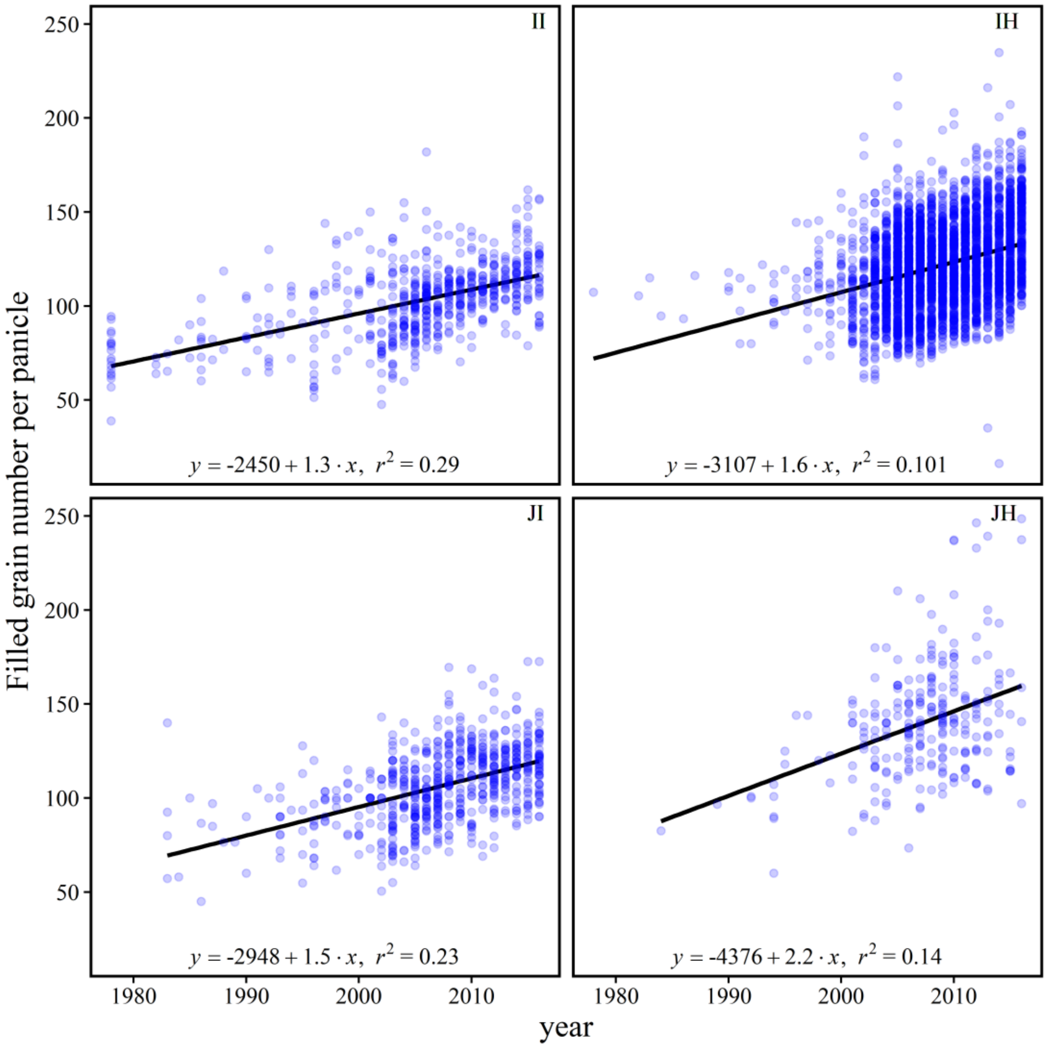

Figure S3. Relationships between year of release and filled grain number per panicle for different rice ecotypes using least-squares regressions. The four ecotypes are: indica inbred (II), and indica hybrid (IH), japonica inbred (JI) and japonica hybrid (JH).

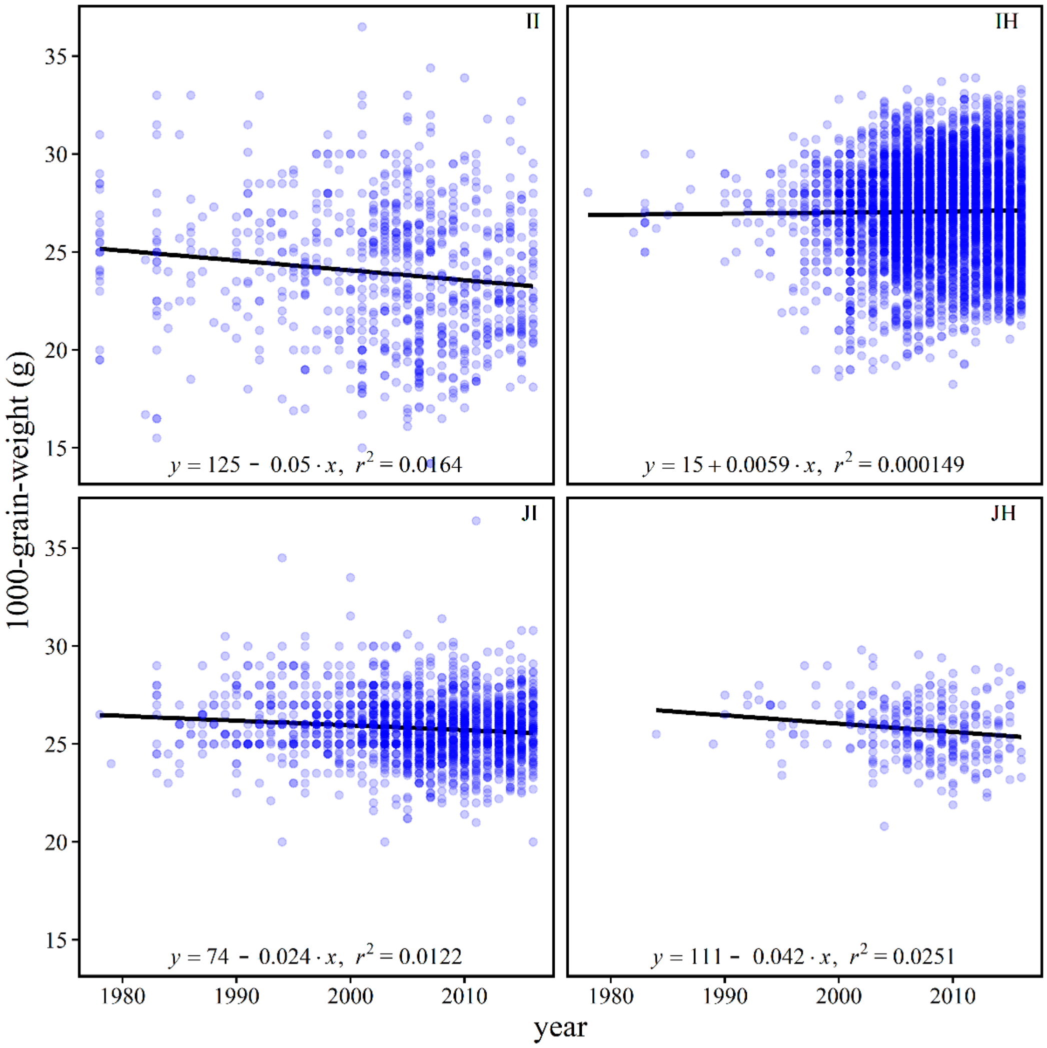

Figure S4. Relationships between year of release and 1000-grain-weight for different rice ecotypes using least-squares regressions. The four ecotypes are: indica inbred (II), and indica hybrid (IH), japonica inbred (JI) and japonica hybrid (JH).

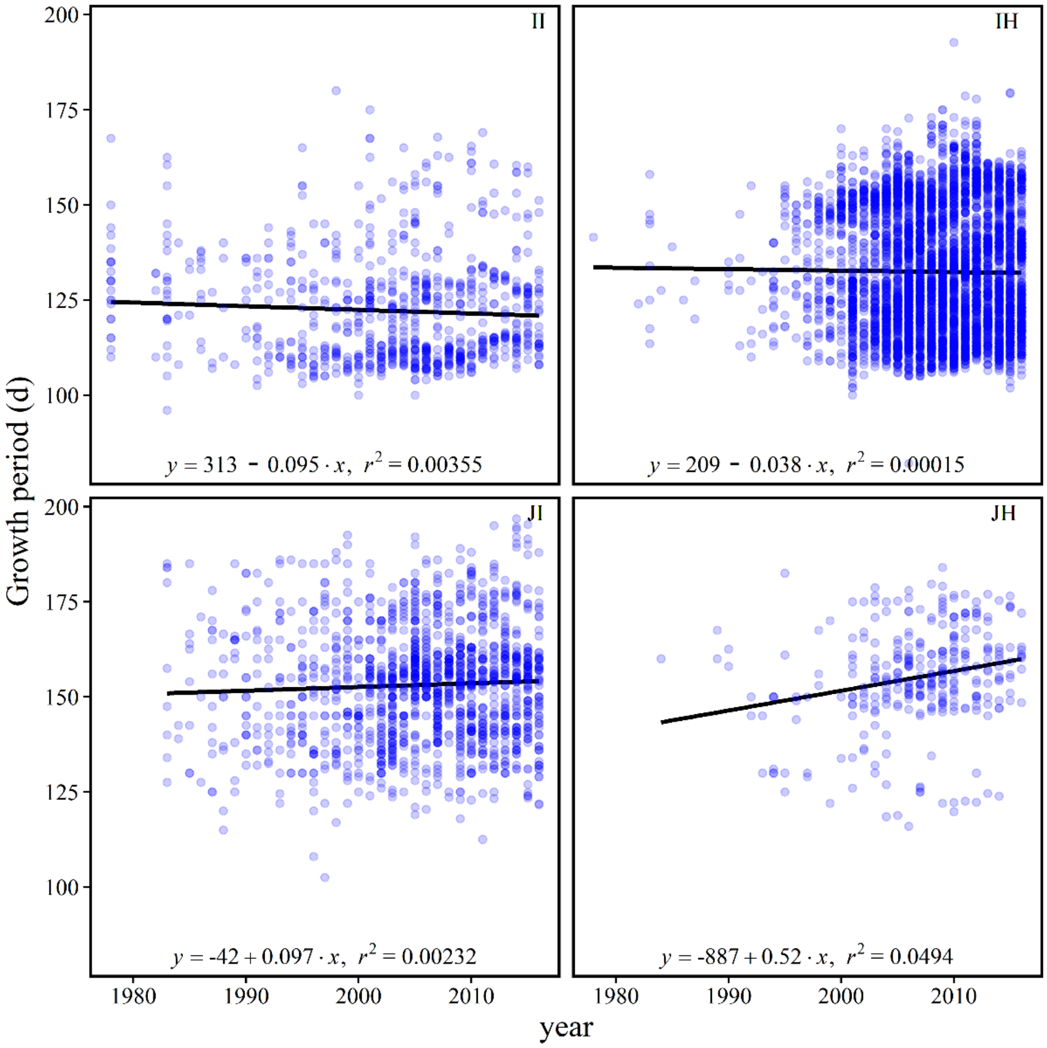

Figure S5. Relationships between year of release and growth period for different rice ecotypes using least-squares regressions. The four ecotypes are: indica inbred (II), and indica hybrid (IH), japonica inbred (JI) and japonica hybrid (JH).

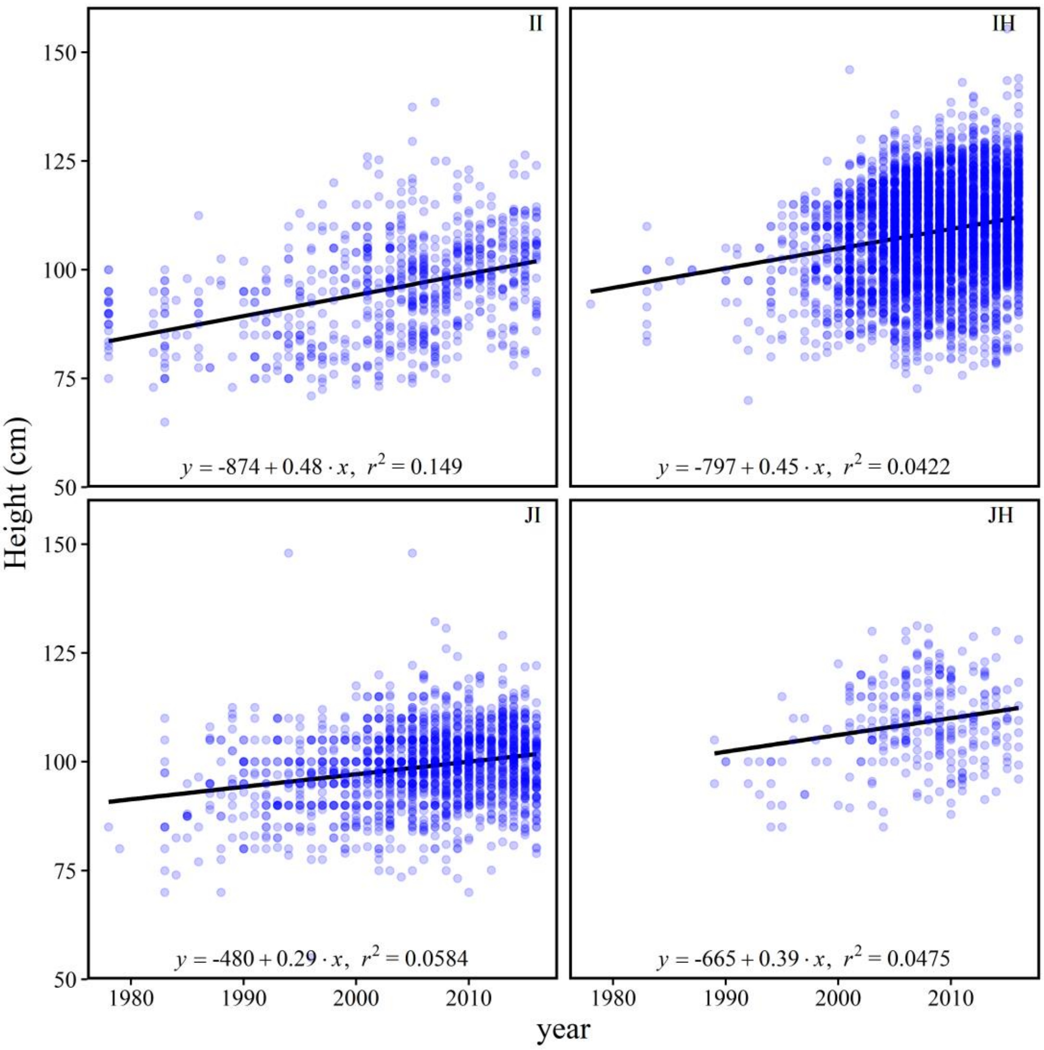

Figure S6. Relationships between year of release and plant height for different rice ecotypes using least-squares regressions. The four ecotypes are: indica inbred (II), and indica hybrid (IH), japonica inbred (JI) and japonica hybrid (JH).

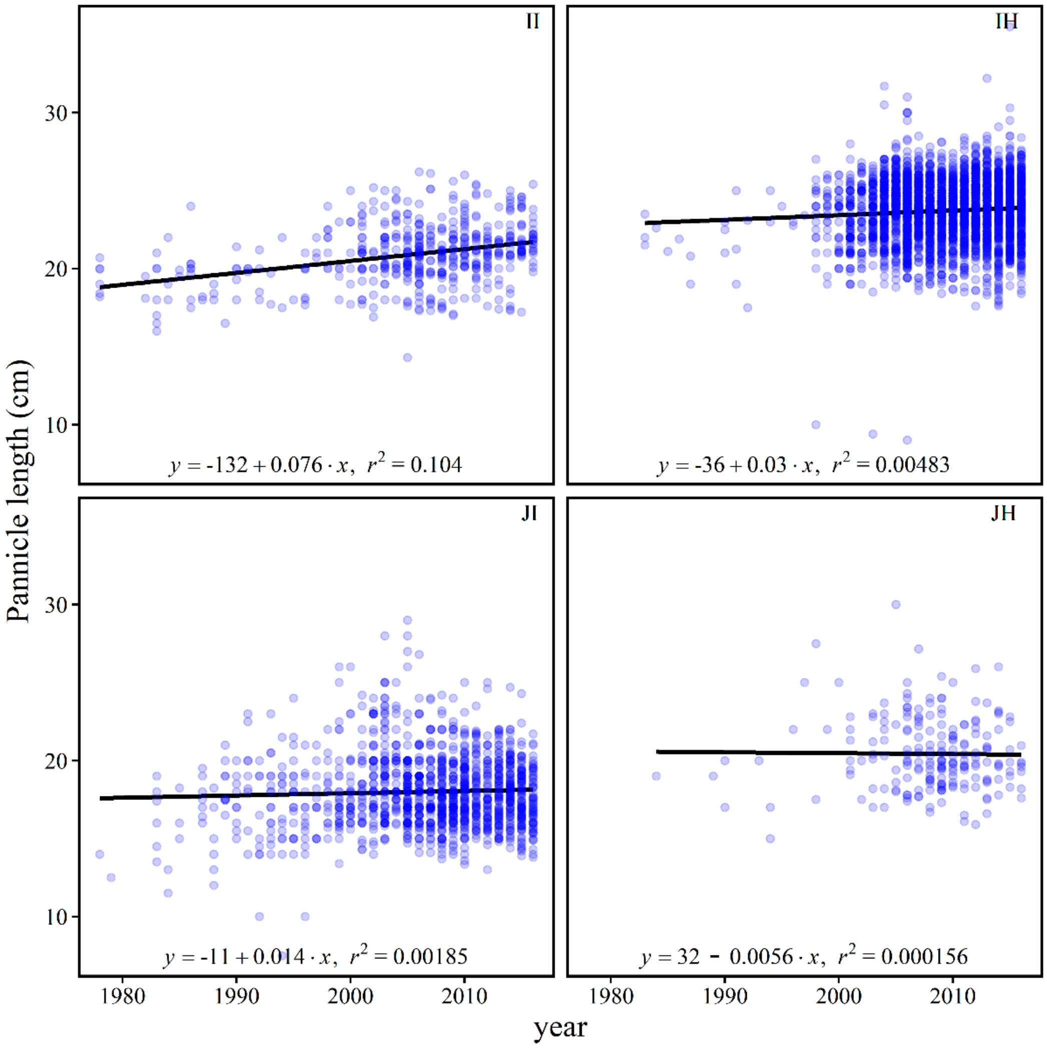

Figure S7. Relationships between year of release and panicle length for different rice ecotypes using least-squares regressions. The four ecotypes are: indica inbred (II), and indica hybrid (IH), japonica inbred (JI) and japonica hybrid (JH).

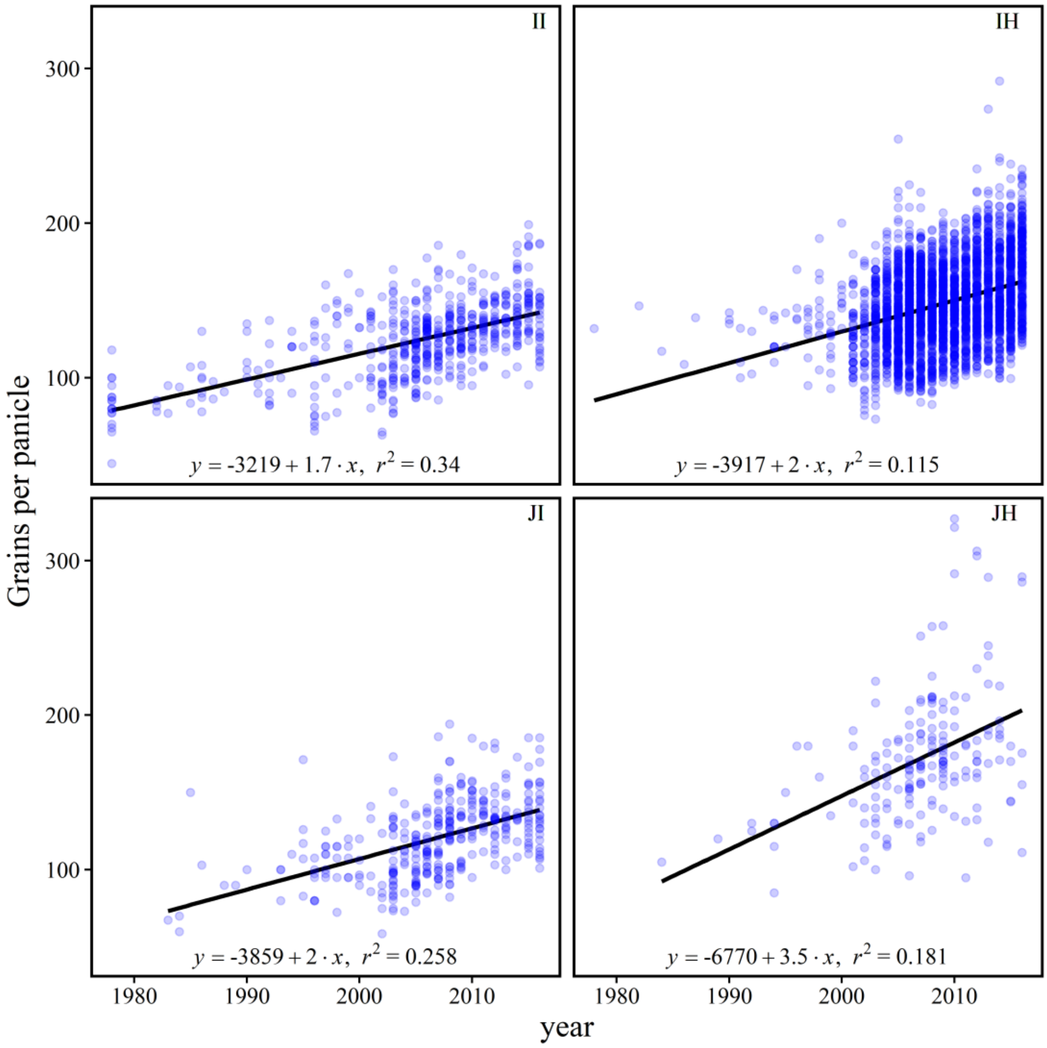

Figure S8. Relationships between year of release and grains per panicle for different rice ecotypes using least-squares regressions. The four ecotypes are: indica inbred (II), and indica hybrid (IH), japonica inbred (JI) and japonica hybrid (JH).

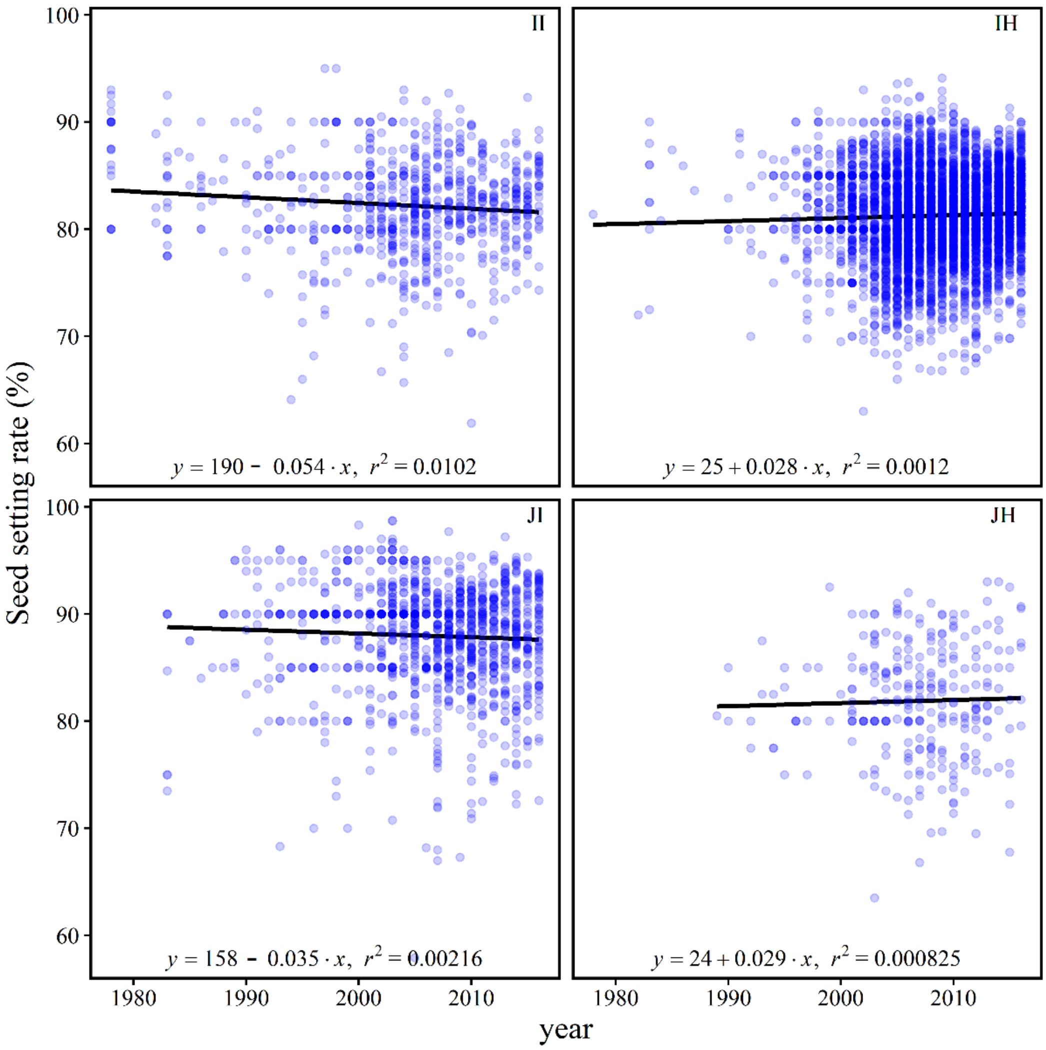

Figure S9. Relationships between year of release and seed setting rate for different rice ecotypes using least-squares regressions. The four ecotypes are: indica inbred (II), and indica hybrid (IH), japonica inbred (JI) and japonica hybrid (JH).

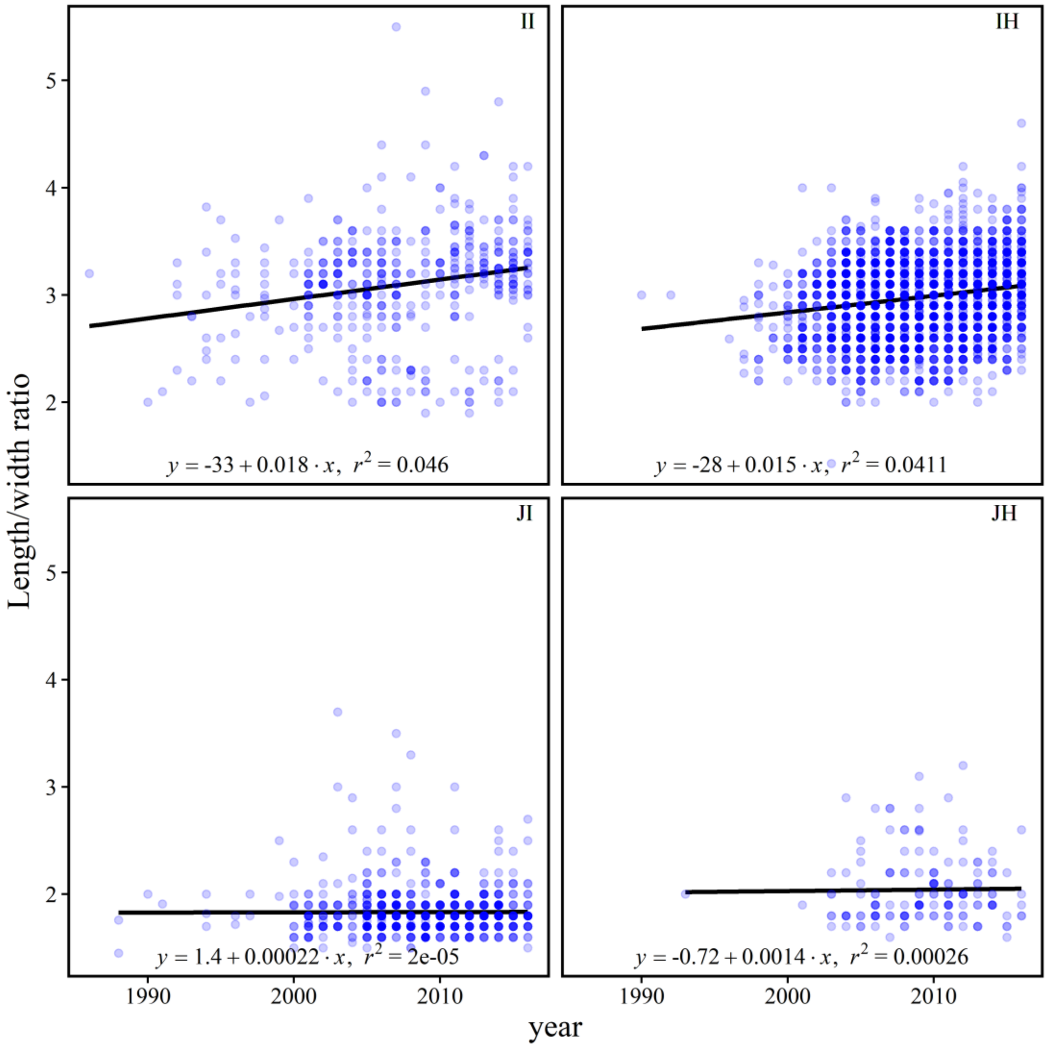

Figure S10. Relationships between year of release and seed length/width ratio for different rice ecotypes using least-squares regressions. The four ecotypes are: indica inbred (II), and indica hybrid (IH), japonica inbred (JI) and japonica hybrid (JH).
